# Usefulness of day 7 lumefantrine plasma concentration as a predictor of malaria treatment outcome in under-fives children treated with artemether-lumefantrine in Tanzania

**DOI:** 10.1186/s12936-020-3150-y

**Published:** 2020-02-11

**Authors:** Manase Kilonzi, Omary Minzi, Ritah Mutagonda, Vito Baraka, Philip Sasi, Eleni Aklillu, Appolinary Kamuhabwa

**Affiliations:** 1 Department of Clinical Pharmacy and Pharmacology, School of Pharmacy, Muhimbili University of Health and Allied Sciences, P. O. BOX 65013, Dar es Salaam, Tanzania; 2 Department of Research, National Institute of Medical Research, Tanga Centre, P O Box 5004, Tanga, Tanzania; 3 Department of Clinical Pharmacology, School of Medicine, Muhimbili University of Health and Allied Sciences, P. O. BOX 6515, Dar es Salaam, Tanzania; 4 Division of Clinical Pharmacology, Department of Laboratory Medicine, Karolinska Institutet, Karolinska University Hospital-Huddinge, C1:68, SE-141 86 Stockholm, Sweden

**Keywords:** Artemether-lumefantrine, Day 7 plasma lumefantrine concentration, Generic, Innovator, Uncomplicated *Plasmodium falciparum*

## Abstract

**Background:**

Day 7 plasma lumefantrine concentration is suggested as a predictor for malaria treatment outcomes and a cut-off of ≥ 200 ng/ml is associated with day 28 cure rate in the general population. However, day 7 lumefantrine plasma concentration can be affected by age, the extent of fever, baseline parasitaemia, and bodyweight. Therefore, this study assessed the usefulness of day 7 lumefantrine plasma concentration as a predictor of malaria treatment outcome in under-fives children treated with generic or innovator drug-containing artemether-lumefantrine (ALu) in Tanzania.

**Methods:**

This study was nested in an equivalence prospective study that aimed at determining the effectiveness of a generic ALu (Artefan^®^) in comparison with the innovator’s product (Coartem^®^). Children with uncomplicated malaria aged 6–59 months were recruited and randomized to receive either generic or innovator’s product. Children were treated with ALu as per World Health Organization recommendations. The clinical and parasitological outcomes were assessed after 28 days of follow up. PCR was performed to distinguish recrudescence and re-infections among children with recurrent malaria. Analysis of day 7 lumefantrine plasma concentration was carried out using a high-performance liquid chromatographic method with UV detection.

**Results:**

The PCR corrected cure rates were 98.7% for children treated with generic and 98.6% for those treated with the innovator product (p = 1.00). The geometric mean (± SD) of day 7 plasma lumefantrine concentration was 159.3 (± 2.4) ng/ml for the generic and 164 (± 2.5) ng/ml for the innovator groups, p = 0.87. Geometric mean (± SD) day 7 lumefantrine plasma concentration between cured and recurrent malaria was not statistically different in both treatment arms [158.5 (± 2.4) vs 100.0 (± 1.5) ng/ml, (p = 0.28) for generic arm and 158.5 (± 2.3) vs 251.2 (± 4.2) ng/ml, (p = 0.24) for innovator arm]. Nutritional status was found to be a determinant of recurrent malaria (adjusted hazardous ratio (95% confidence interval) = 3(1.1–8.2), p = 0.029.

**Conclusion:**

Using the recommended cut-off point of ≥ 200 ng/ml, day 7 plasma lumefantrine concentration failed to predict malaria treatment outcome in children treated with ALu in Tanzania. Further studies are recommended to establish the day 7 plasma lumefantrine concentration cut-off point to predict malaria treatment outcome in children.

## Background

Malaria is still a burden disease in sub-Saharan African countries including Tanzania [1]. The prevalence of the disease in Tanzania is currently decreasing from an average of 18% in 2008 to 7% in 2017 in the general population [2]. Children below 5 years of age and pregnant women are the special populations that are highly affected by malaria. The major strategies implemented in fighting malaria include mass distribution and use of insecticide-treated bed nets (ITNs), indoor residual spraying (IRS), intermittent preventive therapy during pregnancy using sulfadoxine-pyrimethamine (IPTp-SP), seasonal malaria chemoprevention (SMC) and the use of artemisinin-based combination therapy (ACT) for uncomplicated malaria [3].

Children under 5 years of age are the most affected group because of lower immunity against malaria [4]. Partial immunity against malaria is acquired during childhood, therefore, a majority of malaria cases, especially severe malaria with rapid progression to death, occur in young children without acquired immunity [1]. Fever and headache are the common symptoms of uncomplicated malaria in children and sometimes mimic other childhood illnesses particularly gastroenteritis, meningitis/encephalitis, pneumonia and others [5]. Due to low immunity against malaria parasites, malaria in children, if not treated timely, can quickly progress to complicated malaria [5].

ALu is recommended as the first-line anti-malarial drug for the treatment of uncomplicated malaria in mainland Tanzania [6]. The combination is used to treat patients of all age groups including pregnant women, except during the first trimester of pregnancy [7, 8]. Artemisinin derivatives, including artemether, are responsible for the early parasitological response, due to their rapid onset of action [9]. The slowly acting partner drug lumefantrine is responsible for the clearance of the remnant malaria parasites [10, 11]. This means that the therapeutic response of ALu is highly dependent on adequate systemic exposure to lumefantrine, preventing the chance of treatment failures and the emergence of drug-resistant strains [12]. Both artemether and lumefantrine are metabolized by the liver through oxidation by cytochrome P450 enzymes followed by glucuronidation to produce soluble compounds that are excreted [13]. Following several preclinical studies, malaria infection has been proved to interfere with phase I and phase II hepatic metabolism of several anti-malarial drugs, including dihydroartemisinin, primaquine, pyrimethamine, quinine, and quinidine, as well as their biliary excretion as an alternative route [14, 15].

During the introduction of ALu in Tanzania in the year 2006, Coartem^**®**^, which is a product from the innovator company Novartis, was prequalified to be used in the country. Due to poor availability and high cost of innovator’s product, since 2013 generic ACT medicines were approved by the government to be used in the management of uncomplicated malaria [16]. In 2012, a community pharmacy-based study was conducted in Tanzania and found that Artefan^®^, a generic anti-malarial drug-containing ALu was the most widely distributed brand compared to other ACT medicines [16]. However, substandard and fake anti-malarial generic products were previously reported to be available in the drug outlets in South East Asia [17] and sub-Saharan African countries including Tanzania [18–24].

Recently, a survey conducted by the ACT watch Group in Tanzania indicated that the availability of quality-assured ACT medicines was a major concern in most of the drug outlets [25]. Because of the high-level of availability of generic anti-malarial Artefan^®^ in the public health facilities, at the Medical Stores Department (MSD) and in private pharmacies in Tanzania, its bioequivalence with innovator product Coartem^®^ was evaluated in 2012 [16]. Based on the Food and Drug Authority (FDA) criteria of bioequivalence, data did not confirm bioequivalence [16]. Failure of the generic product to fulfill all FDA stipulated criteria for bioequivalence would mean Artefan^®^ was not suitable for use in the management of malaria in clinical settings.

To rule out these doubts and create assurance of the suitability of the generic product, a study was carried out by Kilonzi et al. [26] to assess the malaria treatment outcome of Artefan^®^ in comparison with Coartem^®^. In this study, it was reported that both generic and innovator products are equivalent and effective in the management of uncomplicated malaria in children. The observed findings could be due to adequate drug plasma concentration during treatment which has been linked with good malaria treatment outcomes [12].

Several factors have been reported to affect day 7 plasma concentration. These include adherence, when ALu is taken unsupervised, age, fever on admission, baseline parasitaemia, smoking, body weight, pregnancy and concurrent use of ALu with other medicines [27]. Children below 5 years of age are among the special population with undefined day 7 lumefantrine cut-off point below which recurrent malaria is expected to occur [28]. Studies reported that children (< 5 years) experience lower lumefantrine concentration [27, 29] and among the reasons are bioavailability, the volume of distribution and clearance of drugs which are all age-related [30]. Therefore, this study assessed the usefulness of day 7 lumefantrine plasma concentration (using 200 µg/ml cut-off point) as a predictor of malaria treatment outcome in children under 5 years of age treated with generic or innovator products containing ALu in Tanzania.

## Methods

### Study design, site, and data collection procedures

This study was part of an equivalence prospective study that aimed to determine the effectiveness of anti-malarial generic Artefan^®^ in comparison with the innovator’s product Coartem^®^ [26]. The study was conducted at Kibiti Health Centre in Kibiti district, in the coastal region of Tanzania. In 2019, the prevalence of malaria in Kibiti district was 10.2%. Patients aged between 6 and 59 months with uncomplicated malaria) were enrolled consecutively in the study. After being examined by a physician, screening for malaria positivity was done by malaria Rapid Diagnostic Tests (RDTs). A total of 230 children with confirmed uncomplicated malaria were enrolled in the study. Details on the study population, sample size calculation, sampling technique, inclusion criteria, malaria diagnosis, treatment approach, and laboratory investigations have been described elsewhere [26].

### PCR procedure for the detection of recrudescence and re-infection

PCR procedures to distinguish recrudescence and re-infection was performed according to standard guidelines on methods and techniques for clinical trials on anti-malarial drug efficacy [31, 32] at the National Institute for Medical Research (NIMR), Tanga Centre, Tanzania. DNA was extracted from filter paper (Whatman 3MM) using the QIAamp DNA Mini Kit method (Qiagen GmbH, Hilden, Germany) following the manufacturer’s instructions. Parasites were genotyped for *msp*-*1*, *msp*-*2*, and *glurp* to distinguish between re-infection and recrudescence. Samples were classified as recrudescent infections if there was a complete match of the alleles between day 0 and the day of the recurrent infection while any mismatch was reported as a new infection.

### Day 7 lumefantrine plasma concentration quantification

#### Bioanalytics

On Day 7 from the start of treatment, a venous blood sample (3mls) was collected from the study participant and placed in ethylenediaminetetraacetic acid (EDTA) tube. The sample was then centrifuged for 10 min at 1000–2000*g* to obtain plasma. Plasma was aliquoted into cryotubes and stored at − 20 ℃ at Kibiti Health Centre before being transferred to Muhimbili University of Health and Allied Sciences (MUHAS). The samples were stored in − 80 ℃ freezer at the MUHAS laboratory before analysis. The analysis for determination of lumefantrine was conducted at MUHAS Bioanalytical Laboratory) using the high-performance liquid chromatography (HPLC) method with UV detection as described previously [33].

#### Chromatographic condition

Di-potassium hydrogen phosphate tri-hydrate (4.76 g), distilled water (350 ml) and acetonitrile (650 ml) were used to prepare the mobile phase. The pH was adjusted using ortho-phosphoric acid. LiChrospher 100—RP 18, 5 μm; 5 × 4 mm pre-column and LiChrospher 100—RP18, 5 μm; 125 × 4 mm column were used in the analysis. Quantification of lumefantrine was done at a wavelength of 335 nm, a flow rate of 1.2 ml/min and 20 min run time. Details of the analytical method including selectivity, specificity, calibration, linearity, accuracy, and precision have been reported elsewhere [16, 33].

#### Method validation

Prior to the analysis of patient samples, partial validation of the method was conducted in three inter-day precision and accuracy assay batches. Each run consisted of 8 calibrators in duplicates and four quality controls (QCs) levels at 0.05, 0.1, 1.0 and 8.0 μg/ml. The coefficients of variation (CV%) for quality control samples (low, medium and high) was ≤ 15% and the criteria were applied in all three runs performed on different days.

#### Data analysis

Data were analysed by using statistical package for the social sciences (SPSS) software version 20. Frequency and percentages were used to summarize participants’ demographic characteristics. Independent student’s t-test was used to assess differences in the geometric mean for log-transformed day 7 plasma lumefantrine concentrations between individuals with recurrent malaria and those who were cured within treatment groups. A cut-off point of 200 µg/ml of day 7 plasma lumefantrine concentration was used during analysis. Linear regression analysis was used to assess determinants of day 7 lumefantrine concentrations in children treated with ALu. Cox regression analysis was used to determine the factors associated with recurrent malaria following treatment with ALu. P-value < 0.05 was considered as significant.

## Results

Venous blood for quantification of day 7 lumefantrine concentration was obtained from 147 study participants. Of these, 76 had been randomized to use the generic product (Artefan^®^) and 71 were given the innovator product (Coartem^®^). Due to the inadequate amount of plasma obtained, 21 participants in the generic and 22 in the innovator’s arms of treatment were excluded from plasma lumefantrine quantification and subsequent data analysis.

### Characteristics of study participants

The majority of study participants were aged ≤ 24 months in the generic (62.5%) and > 24 months in innovator (53.1%) product treatment arms. Females were the majority (57.1%) in the generic treatment arm, while males were 50.9% in the innovator’s treatment arm. Most (52.9% on generic and 47.1% on innovator arms of treatment) of the study participants had baseline temperature of ≥ 37.5 ℃ and the majority had baseline parasitaemia of between 1000/µl and 10,000/µl in innovator’s treatment arm (70.8%) and ≥ 10,000/µl in the generic arm (57.9%), p = 0.025 (Table 1).

**Table 1:** Study participants’ characteristics (n = 104)

Variables	Drug given		p-value
	Generic n (%)	Innovator n (%)	
Age (months)			
≤ 24	25 (62.5%)	15 (37.5%)	0.12
> 24	30 (46.9%)	34 (53.1%)	
Sex			
Female	28 (57.1%)	21 (42.9%)	0.41
Male	27 (49.1%)	28 (50.9%)	
Weight (kg)			
< 15	39 (50.6%)	38 (49.4%)	0.44
≥ 15	16 (59.3%)	11 (40.7%)	
Baseline temperature			
< 37.5 ℃	9 (52.9%)	8 (47.1%)	0.99
≥ 37.5 ℃	46 (52.9%)	41 (47.1%)	
Baseline hemoglobin (g/dl)			
< 11	41 (56.9%)	31 (43.1%)	0.30
≥ 11	12 (48%)	13 (52%)	
Baseline parasitaemia (/µl)			
< 1000	15 (65.2%)	8 (34.8%)	
1000–10,000	7 (29.2%)	17 (70.8%)	
≥ 10,000	33 (57.9%)	24 (42.1%)	0.025

### Day 28 malaria treatment outcome

The PCR adjusted adequate clinical and parasitological response (ACPR) was 98.6% for innovator and 98.7% for generic treatment arms (p = 1.00. There was only one participant in each treatment arm with recrudescence parasitaemia on day 28. Although the difference in ACPR was not statistically significant between the two treatment arms, re-infection was more prevalent in children treated with the innovator (8.5%) than in the generic (5.3%) arms of treatment, p = 0.76 (Table 2).

**Table 2:** Day 28 malaria treatment outcome (n = 147)

Variables	Day 28 treatment outcomes		p-value
	Generic n (%)	Innovator n (%)	
Adequate clinical and parasitological response (ACPR)	71 (93.4)	64 (90.1)	0.76
Re-infection	4 (5.3)	6 (8.5)	
Recrudescence	1 (1.3)	1 (1.4)	
PCR corrected cure rate	75 (98.7)	70 (98.6)	1.00

### Day 7 plasma lumefantrine concentration and day 28 malaria treatment outcome

Overall geometric mean (± SD) of day 7 plasma lumefantrine concentration were 160.3 (± 2.4) for the cured and 173.4 (± 3.3) for those with recurrent malaria (p = 0.79). Majority of study participants had day 7 lumefantrine concentration of < 200 ng/ml in both treatment groups, 31 (56.4%) for generic and 25 (51%) for innovator products (p = 0.59). The geometric mean (± SD) of day 7 plasma lumefantrine concentrations were 159.3 (± 2.4) ng/ml for the generic and 164 (± 2.5) ng/ml for the innovator groups (p = 0.87). Geometric mean (± SD) day 7 lumefantrine plasma concentration between cured and recurrent malaria was not statistically different in both treatment arms [158.5 (± 2.4) vs 100.0 (± 1.5) ng/ml, (p = 0.28) for generic arm and 158.5 (± 2.3) vs 251.2 (± 4.2) ng/ml, (p = 0.24) for innovator arm].

### Determinants of day 7 lumefantrine plasma concentrations in children treated with ALu

A linear regression analysis was conducted to determine factors associated with day 7 lumefantrine plasma concentration for children treated with ALu. The factors included in the analysis were gender, age, body weight, mid-upper arm circumference (MUAC), baseline temperature (fever on admission), baseline haemoglobin and parasitaemia. None of the variables was associated with day 7 plasma lumefantrine concentration.

### Factors determining recurrent malaria in children treated with ALu

Several variables were analysed against treatment outcome to assess determinants of recurrent malaria in children under 5 years of age treated with ALu. Factors assessed were gender, age, weight, baseline temperature, baseline parasitaemia, baseline haemoglobin, nutritional status (MUAC) and day 7 lumefantrine concentration. Only nutritional status (MUAC) was found to be a determinant of recurrent malaria on multivariate analysis with adjusted hazardous ratio (95% confidence interval) of 3 (1.1–8.2) at a p-value of 0.029 (Table 3).

**Table 3:** Cox regression analysis showing determinants of recurrent malaria among children (< 5 years) treated with Alu. (n = 147)

Variables	cHRs (95% CI)	p value	aHRs (95% CI)	p value
Age (months)	1 (0.98–1.05)	0.46	1.3 (0.9–2.0)	0.16
Weight (kg)	1.1 (0.95–1.42)	0.16	0.1 (0.0–1.4)	0.09
WAZ	1.7 (0.94–3.1)	0.08	31 (0.6–1577)	0.09
MUAC	1.4 (0.89–2.1)	0.16	3 (1.1–8.2)	0.029
Baseline temperature (℃)	1.0 (0.64–1.7)	0.85	1.0 (0.5–1.9)	0.94
Baseline parasitemia (g/dl)				
< 1000	0.0 (0.0–)	0.98	0.0 (0.00–)	0.96
1000–10,000	0.9 (0.2–3.2)	0.83	0.7 (0.2–3.5)	0.69
≥ 10,000	1		1	
Sex				
Female	0.7 (0.2–2.3)	0.58	1.2 (0.3–5.2)	0.82
Male	1			
Treatment arm				
Artefan^®^	0.7 (0.2–2.1)	0.47	0.5 (0.1–2.3)	0.57
Coartem^®^	1		1	
Baseline hemoglobin (g/dl)	1.2 (0.8–1.6)	0.34	1.3 (0.8–2.0)	0.24
Day 7 plasma lumefantrine concentration (ng/ml) (n = 104)	1.2 (0.24–5.9)	0.83	1.3 (0.2–8.1)	0.81

## Discussion

This study assessed day 7 lumefantrine plasma concentration as a predictor of malaria treatment outcome in under-fives children treated with generic and innovator products containing ALu in Tanzania. The findings of this study indicate that the majority of children had day 7 plasma lumefantrine plasma concentrations below the recommended ≥ 200 ng/ml cut-off point [27] in both generic and innovator’s arm. This finding is consistent with what has been reported recently in Uganda [34] and by WorldWide Antimalarial Resistance Network (WWARN) 2015 report, in which day 7 plasma concentration in children below 5 years of age were reported to be low compared to the general population [27]. The low day 7 lumefantrine concentration observed in this study could be explained by the poor pharmacokinetic parameters in children.

Developmental changes in absorptive surfaces in children’s gastrointestinal tract affect the rate and extent of bioavailability of peroral medications, such as ALu [30]. Besides, maturation of metabolizing enzymes including cytochrome P450 occurs as a child grow, and this has also been associated with the observed poor pharmacokinetics of drugs in children [11, 30]. Nevertheless, this trial was unsupervised anti-malarial treatment whereby only the first dose was administered at the clinic and the rest were taken at home. Administering medicines to sick children is very difficult and vomiting is the major challenge. Hence the observed low day 7 plasma lumefantrine concentration could be contributed by poor adherence as previously reported in Uganda in a study on supervised *versus* unsupervised anti-malarial treatment with six-dose ALu [38].

Despite the low day 7 plasma concentration observed in both generic and innovator arms of treatment, day 28 PCR corrected malaria treatment outcome observed was excellent. The finding provides assurances of using the generic as well as innovator anti-malarial products in the management of uncomplicated malaria in children despite the bioinequivalent results reported by Minzi et al. [16]. Detailed information on the therapeutic outcomes between generic and innovator anti-malarial product when used in the management of uncomplicated malaria has been described elsewhere [26]. The observed high cure rates in this study are comparable to a 95% cure rate which was reported in Ethiopia among patients treated with ALu for management of uncomplicated malaria [35, 36].

The good treatment outcome observed in this study could be due to the activity of the active metabolite of lumefantrine (desbutyl-lumefantrine) [37]. Indeed, studies have reported that desbutyl-lumefantrine (DBL) has greater anti-malarial potency and mildly synergistic effect with dihydroartemisinin in vitro [38, 39]. Furthermore, a study of day 7 plasma concentration of the metabolite and adequate clinical and parasitological response suggests that DBL could be used as an alternative to lumefantrine as part of ACT programme [37].

In the current study, most of the observed recurrent malaria was due to re-infection and only 1 recrudescence was observed in each group of treatment arms on day 28. The observed small number of recrudescence indicates that the artemisinin derivative (artemether) is still effective in Tanzania. Recrudescence after being treated with ALu is highly observed in areas with reported emergence of artemisinin resistance [27, 40]. This study was conducted in the coastal region of Tanzania where there are no reports of resistance of *Plasmodium* species to artemisinins, therefore supporting the observed high cure rate. Despite these findings, it was observed that there were re-infections in some children. This could be explained by the endemicity of malaria in the study area as well as the vulnerability of children under 5 years of age, due to inadequate immunity to malaria [1, 4].

Linear regression analysis indicated that age, gender, weight, and MUAC, baseline haemoglobin, temperature, and parasitaemia have no association with day 7 plasma lumefantrine concentration. These results are contrary to the findings from the general population where factors such as baseline parasitaemia, unsupervised administration of ALu, and baseline haemoglobin levels have been reported to affect day 7 plasma concentration and malaria treatment outcome [27].

In this study, weight, baseline temperature, baseline parasitaemia, baseline haemoglobin, day 7 plasma lumefantrine concentration and nutritional status (MUAC) were assessed against recurrent malaria using Cox regression analysis. All factors except nutritional status were not determinants of recurrent malaria. This finding is in line with previously reported observations in which children with malnutrition were found to be at a high risk of acquiring malaria infection [41]. Malnutrition weakens immunity development [42, 43] and is probably one of the reasons why children with poor nutritional status suffer recurrent malaria after treatment with ALu.

## Limitations

The sample size calculation did not take into account different age groups for children aged < 5 years. This probably impaired the power of the study to establish the effect of age on day 7 plasma lumefantrine concentration and treatment outcome in young children. Because it takes up to 2 years for children to acquire premunition immunity against malaria following several exposures, the larger sample size is recommended in future studies. The trial was unsupervised and parents/guardians were asked to bring medicine blister packs with children on day 3 for assessment of adherence. Due to limitations of pill count as a method for assessment of adherence, it could not be verified with certainty whether all children were administered ALu tablets by parents or guardians as prescribed. In addition, the nutritional status of the study participants was measured using MUAC only.

## Conclusion

Using the recommended cut-off point of ≥ 200 ng/ml, day 7 plasma lumefantrine concentration failed to predict malaria treatment outcome in children treated with generic or innovator products containing ALu for uncomplicated malaria in Tanzania. Nevertheless, day 7 plasma lumefantrine concentration levels were comparable in both generic and innovator product treatment arms. The study to establish the day 7 plasma lumefantrine concentration cut-off point to predict malaria treatment outcome in children, as well as the role of DBL in the treatment of malaria, is recommended. Taken together, the findings of this study indicate that the use of ALu generic products should be continued for the management of uncomplicated malaria in children in Tanzania.

## Data Availability

The datasets used and/or analysed during the current study are available from the corresponding author on reasonable request.

## Abbreviations

ACPR: Adequate clinical and parasitological response
ACTs: Artemisinin combination therapies
ALu: Artemether-lumefantrine
AUC: Area under the concentration–time curve
BE: Bioequivalence
Cmax: Maximum plasma concentration
CV: Coefficients of variation
DBL: Desbutyl-lumefantrine
DNA: Deoxyribonucleic acid
EDTA: Ethylenediaminetetraacetic acid
FDA: Food and drug authority
HPLC: High-performance liquid chromatography
IPTP –SP: Sulfadoxine-pyrimethamine
IRS: Indoor residual spraying
ITNs: Insecticide-treated bed nets
MS: Microsoft
MSD: Medical stores department
MSP: Merozoite surface protein
MUAC: Mid-upper arm circumference
MUHAS: Muhimbili University of Health and Allied Sciences
NIMR: National Institute of Medical Research
NTD: Neglected tropical diseases
PCR: Polymerase chain reaction
RDT: Rapid diagnostic tests
SD: Standard deviation
SMC: Seasonal malaria chemoprevention
SPSS: Statistical package for social sciences
SSA: Sub-Saharan African
UV: Ultraviolet
WHO: World Health Organization
WWARN: World Wide Antimalarial Resistance Network
